# Rational Engineering of a Cold-Adapted α-Amylase from the Antarctic Ciliate Euplotes focardii for Simultaneous Improvement of Thermostability and Catalytic Activity

**DOI:** 10.1128/AEM.00449-17

**Published:** 2017-06-16

**Authors:** Guang Yang, Hua Yao, Matteo Mozzicafreddo, Patrizia Ballarini, Sandra Pucciarelli, Cristina Miceli

**Affiliations:** a School of Biosciences and Veterinary Medicine, University of Camerino, Camerino, Macerata, Italy; b Department of Medicinal Chemistry, University of Florida, Gainesville, Florida, USA; North Carolina State University

**Keywords:** Antarctica, cold active molecule, environmental adaptation, hydrolytic activity, marine microbiology, mutant, thermostability

## Abstract

The α-amylases are endo-acting enzymes that hydrolyze starch by randomly cleaving the 1,4-α-d-glucosidic linkages between the adjacent glucose units in a linear amylose chain. They have significant advantages in a wide range of applications, particularly in the food industry. The eukaryotic α-amylase isolated from the Antarctic ciliated protozoon Euplotes focardii (*Ef*Amy) is an alkaline enzyme, different from most of the α-amylases characterized so far. Furthermore, *Ef*Amy has the characteristics of a psychrophilic α-amylase, such as the highest hydrolytic activity at a low temperature and high thermolability, which is the major drawback of cold-active enzymes in industrial applications. In this work, we applied site-directed mutagenesis combined with rational design to generate a cold-active *Ef*Amy with improved thermostability and catalytic efficiency at low temperatures. We engineered two *Ef*Amy mutants. In one mutant, we introduced Pro residues on the A and B domains in surface loops. In the second mutant, we changed Val residues to Thr close to the catalytic site. The aim of these substitutions was to rigidify the molecular structure of the enzyme. Furthermore, we also analyzed mutants containing these combined substitutions. Biochemical enzymatic assays of engineered versions of *Ef*Amy revealed that the combination of mutations at the surface loops increased the thermostability and catalytic efficiency of the enzyme. The possible mechanisms responsible for the changes in the biochemical properties are discussed by analyzing the three-dimensional structural model.

**IMPORTANCE** Cold-adapted enzymes have high specific activity at low and moderate temperatures, a property that can be extremely useful in various applications as it implies a reduction in energy consumption during the catalyzed reaction. However, the concurrent high thermolability of cold-adapted enzymes often limits their applications in industrial processes. The α-amylase from the psychrophilic Antarctic ciliate Euplotes focardii (named *Ef*Amy) is a cold-adapted enzyme with optimal catalytic activity in an alkaline environment. These unique features distinguish it from most α-amylases characterized so far. In this work, we engineered a novel *Ef*Amy with improved thermostability, substrate binding affinity, and catalytic efficiency to various extents, without impacting its pH preference. These characteristics can be considered important properties for use in the food, detergent, and textile industries and in other industrial applications. The enzyme engineering strategy developed in this study may also provide useful knowledge for future optimization of molecules to be used in particular industrial applications.

## INTRODUCTION

The α-amylases (Enzyme Commission [EC] no. 3.2.1.1) are endo-acting enzymes that hydrolyze starch by randomly cleaving the 1,4-α-d-glucosidic linkages between the adjacent glucose units in a linear amylose chain (1, 2). On the basis of sequence similarity, α-amylases have been classified into the glycoside hydrolase (GH) families (3), mostly incorporated into the GH13 family in the carbohydrate-active enzymes (CAZy) database (4). However, with the rapidly increasing number of α-amylases recently discovered, they have also been identified as representatives of other GH families (3). The evolutionary relatedness among members of the GH13 family has been recently extensively revised, and the family is presently subdivided into 42 subfamilies on the basis of similarities in their sequences (5). The tertiary structure of each of these enzymes is characterized by a (β/α)_8_ barrel containing a conserved catalytic triad (Asp, Glu, and Asp) that forms the active site (6).

α-Amylases have been utilized in a wide range of industrial processes, such as in food, detergent, textile, and paper industries (7, 8). They represent approximately 30% of the world's enzyme market (2). α-Amylases can be obtained from several sources, including plants, animals, and microorganisms. Microbial α-amylases are generally attractive for biotechnological and industrial applications (1, 9), particularly those produced by extremophiles, as they can withstand harsh conditions (10–15). In general, cold-adapted enzymes produced by psychrophiles possess high biotechnological value and provide economic benefit, being more productive at low temperatures than mesophilic or thermophilic homologs. This implies energy savings in industrial processes (16, 17). However, although a few number of cold-adapted α-amylases have been studied to understand the molecular adaptation of cold-adapted enzymes (18–22), they are very rarely developed for immediate use in industrial applications (23). As a matter of fact, there has been a high demand for the discovery of a novel cold-adapted α-amylase for use in various industrial processes (24).

Cold-adapted enzymes possess a range of structural features that confer higher structural flexibility than thermostable homologs, which usually translates to high specific activity at low temperatures (25). However, an increased flexibility may also represent a two-edged sword for psychrophilic organisms, since it also increases the likelihood that the proteins may undergo denaturation in response to small changes in temperature (26). Therefore, the high thermolability of cold-adapted enzymes has been a major drawback for their use in industrial applications (17, 27). One way to solve this problem is to modify existing cold-adapted enzymes by site-directed mutagenesis combined with a rational design approach (28), which generally is based on structure-guided consensus sequence alignments with sequences that have moderate or high amino acid identity to reduce the number of possible target residues to be mutated. Previously, rational design principles were successfully utilized to enhance the stability and/or catalytic efficiency of enzymes such as maltogenic amylase and patatin-like phospholipase (29–31). These changes reduced the molecular flexibility of the polypeptide by (i) introducing hydrophobic residues in the protein core, (ii) introducing disulfide and salt bridges to increase electrostatic interactions in the polypeptide, (iii) stabilizing α-helix dipoles, (iv) introducing Pro residues, and (v) loop shortening to decrease loop entropy (28, 32).

Recently, the cold-adapted α-amylase from the Antarctic ciliate Euplotes focardii, named *Ef*Amy, was heterologously expressed in Escherichia coli and biochemically characterized (33). The ciliated protozoon E. focardii shows a strictly psychrophilic phenotype (34, 35). The amylolytic activity of *Ef*Amy was compared with that of the homologous enzyme, *Ec*Amy, from the mesophilic congeneric species E. crassus (33). The results showed that *Ef*Amy represents a classical psychrophilic enzyme with high hydrolytic activity at low temperatures (5 to 25°C) and high thermolability. Furthermore, *Ec*Amy displayed a 2-fold increased thermostability at 50°C compared with that of *Ef*Amy. In this study, we applied site-directed mutagenesis to generate a cold-active *Ef*Amy with improved thermostability and catalytic efficiency at low temperatures. We applied a rational design approach to identify residues that contribute to the structural stability and catalytic efficiency of the cold-active *Ef*Amy α-amylase. Biochemical assays of engineered versions of *Ef*Amy revealed that the combination of mutations at the surface loops and catalytic core resulted in significant increases in thermostability and catalytic efficiency of the enzyme. The novel α-amylases generated in our study could have important applications in the detergent and food and beverage industries, where the cold-adapted enzymes have already been widely applied and have even led to the evolution of the conventional processes (36). Furthermore, the success of our rational design strategy suggests that it may be universally applied to other enzymes.

## RESULTS AND DISCUSSION

### *Ef*Amy sequence analysis.

**FIG 1:**
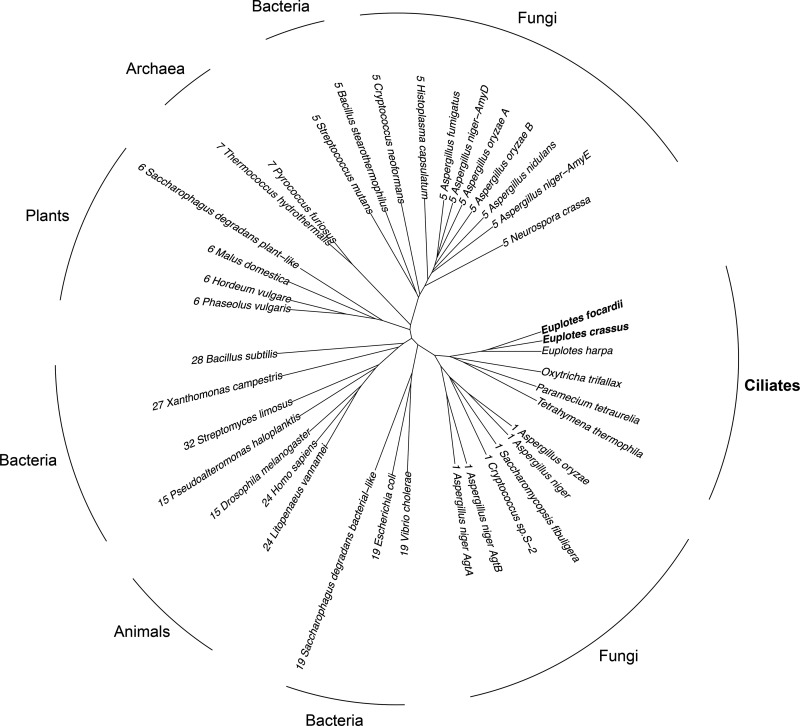
Phylogenetic tree of GH13 family enzymes from a variety of organisms, including ciliates. Each amylase sequence is indicated by the name of the species that produces it preceded by the number of the GH13 subfamily and followed by letters that label different amylase forms from the same species when present. GH13 subfamilies are attributed according to van der Kaaij et al. (81) and Janeček and Gabriško (5). Database accession numbers for the amylases are reported in Table S2 in the supplemental material.

In a previous study (33), we biochemically characterized two homologous α-amylases from two closely related Euplotes species, the psychrophilic E. focardii (named *Ef*Amy) and the mesophilic E. crassus (named *Ec*Amy). We showed that *Ef*Amy and *Ec*Amy share a relatively high sequence similarity (68% identity, 82% similarity). To identify their evolutionary relations with other members of the GH13 subfamilies, *Ef*Amy and *Ec*Amy were aligned with α-amylases from representative taxa of Bacteria, Archaea, and Eukarya. The alignment was then converted into a phylogenetic tree (Fig. 1). The tree clearly shows that *Ef*Amy (named in the tree as Euplotes focardii) and *Ec*Amy (named Euplotes crassus) together with homologous sequences from other ciliates, such as Euplotes harpa, Oxytricha trifallax, Tetrahymena thermophila, and Paramecium tetraurelia, cluster within the GH13_1 subfamily clade that is represented by several well-studied fungus and yeast α-amylases, including Aspergillus niger acid α-amylase (37), Aspergillus oryzae TAKA α-amylase (38), and Saccharomycopsis fibuligera α-amylase (39).

### Rational design of *Ef*Amy mutations.

**FIG 2:**
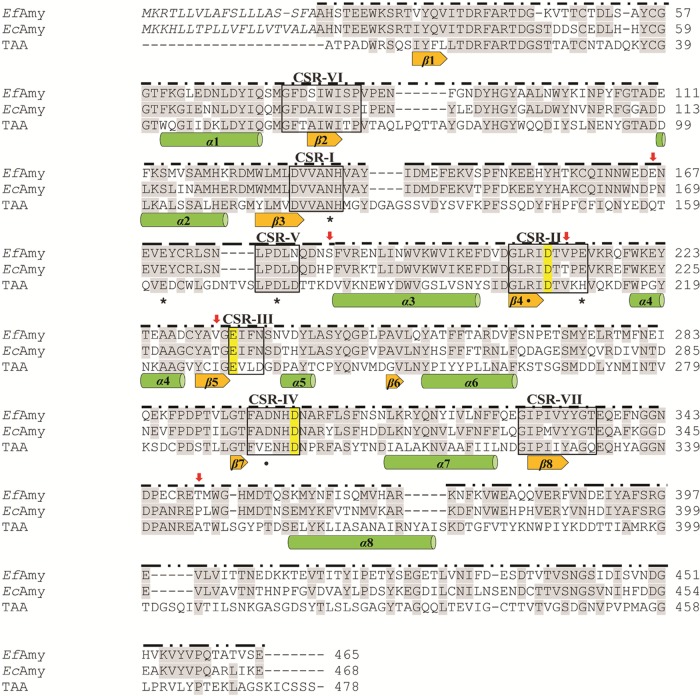
Euplotes α-amylase sequence analysis. Alignment of the predicted amino acid sequences of *Ef*Amy and *Ec*Amy with the homologous A. oryzae TAKA α-amylase A (TAA) that was included due to available molecular structures used in the homology model shown in Fig. 3. Conserved residues are lightly shaded. The α-amylase catalytic residues of the Asp, Glu, and Asp triad are marked in yellow. The five mutation sites are labeled with red arrows. The seven conserved α-amylase sequence regions are boxed. The amino acids of putative signal peptides are in italics. Secondary structure elements are presented below the sequences, which correspond to the TAA α/β barrel, as described by Machius et al. (82). TAA residues involved in Ca^2+^ (*) and Cl^−^ (**•**) binding (82) are indicated below the sequences. Sequence regions belonging to domains A (**– · –**), B (**– – –**), and C (**– · · –**) are labeled above the sequences.

Previous findings from the biochemical characterization of *Ef*Amy and *Ec*Amy indicate significant differences in the catalytic properties of the two enzymes, such as in their thermostability and temperature-dependent catalytic efficiency (33). In this study, we combined rational design and site-directed mutagenesis approaches to modify *Ef*Amy to develop novel α-amylases that are both thermostable and highly active at low temperatures. As the first step of our strategy, we performed a detailed sequence analysis of the cold-adapted *Ef*Amy with the homologous mesophilic *Ec*Amy (Fig. 2). This included a multiple-sequence alignment (MSA) and protein secondary structure analysis. In the MSA, the TAKA α-amylase A (TAA) from A. oryzae was also included, since the three-dimensional protein structure of this enzyme was used as the template for homology modeling of Euplotes α-amylases in the subsequent analysis. The overall low conservation of the protein sequences among GH13 family enzymes prevented the inclusion of additional α-amylases in the alignment (40). The purpose of this analysis was to compare the sequences of two homologous enzymes from closely related species to identify the residues possibly responsible for their distinguished catalytic properties revealed by the previous biochemical characterization (33). In general terms, the conservation between *Ef*Amy and *Ec*Amy was not uniform along the entire sequence. The highest amino acid conservation level was found at the sequences corresponding to the A, B, and C domains (Fig. 2), which showed 71, 81, and 53% identity, respectively. Subsequently, for the selection of the mutation sites, we focused our attention on the A and B domains, since changes in the C domain appear to have no effects on α-amylase thermostability and activity (41).

**FIG 3:**
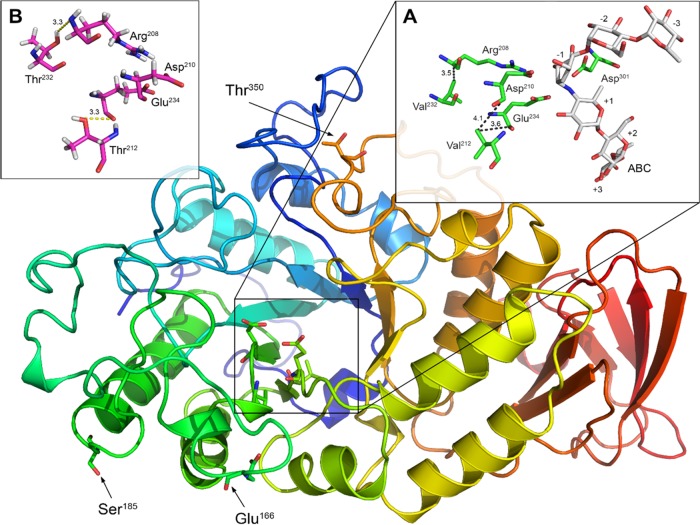
Low-resolution homology model of *Ef*Amy. *Ef*Amy residues that were chosen for mutation to Pro are indicated in black. (A) The catalytic dyad (Glu234 and Asp210) and the other residues involved in the interaction with the ligand ABC (reporting the accepted nomenclature from +3 to −3; see Materials and Methods and reference^80^). Feasible nonpolar interactions indicated with dotted lines and their lengths in Å are also reported. (B) The same region shown in panel A with V212T and V232T mutations. The most likely formed hydrogen bonds are indicated by dotted lines with their lengths in Å.

Through an intensive analysis of the sequences, we detected three substitutions in the A and B domains of *Ef*Amy and *Ec*Amy, i.e., E166P, S185P, and T350P (where each number refers to the sequence position that is involved in the substitution and the letters that precede and follow the number stand for amino acids present in E. focardii and E. crassus, respectively). It is unsurprising that a higher number of Pro residues is observed in the mesophilic α-amylase than in the psychrophilic *Ef*Amy. In fact, Pro avoidance has been generally assumed as the molecular mechanism of cold adaptation of the psychrophilic enzymes, since Pro residues in loops are supposed to infer increased rigidity to the polypeptide and a smaller number of Pro residues has been noted in loops connecting secondary structures in several cold-adapted enzymes investigated so far (42). Very intriguingly, we observed through structural modeling analysis that the three Pro substitutions between *Ef*Amy and *Ec*Amy were all located in the surface loops (Fig. 3) (i.e., two of them were found in the loop of the B domain and another was found in the loop of the A domain), which have been reported to be of major importance to the overall stability of α-amylases (7). Therefore, we primarily chose to mutate the three residues in the loops of *Ef*Amy (Glu166, Ser185, and Thr350) to Pro to achieve an increased thermostability of the cold-adapted enzyme.

Through sequence alignment and modeling analysis, we also observed that Val212 and Val232 in *Ef*Amy were replaced by Thr in *Ec*Amy. As shown in Fig. 3, Val212 is located in close proximity to the catalytic residues, Asp210 (at a distance of approximately 4.1 Å) and Glu234 (at a distance of approximately 3.6 Å), while Val232 is located in proximity to residue Arg208 (at a distance of approximately 3.5 Å), which according to Brzozowski and Davies (43) plays a role in the stabilization of ligand (e.g., with ligand ABC as shown in Fig. 3A). Val212 and Val232 interact with the catalytic residues by nonpolar interactions as shown in Fig. 3A. Previous studies have indicated that the modification of amino acids surrounding the catalytic sites of enzymes will significantly affect the catalytic activity and stability (31, 44). In our case, the replacement of these residues with polar amino acids, such as Thr (as occurs in *Ec*Amy), most likely will produce the establishment of H bonds between the two Thr residues and Asp and Arg residues of the catalytic site as predicted and shown in Fig. 3B. The predicted shorter length and, consequently, the higher strength of these hydrogen bonds with respect to the nonpolar interactions (which, moreover, become weaker under cold temperature conditions [45]) may confer higher stability versus the ligand and better catalytic activity of the enzyme. Therefore, Val212 and Val232, located around the active sites of *Ef*Amy, were chosen for mutagenesis to probe their effects on the catalytic efficiency and thermostability of *Ef*Amy. In addition to the single mutations, variants containing these substitutions combined (V212T/V232T, E166P/S185P/T350P, and E166P/S185P/T350P/V212T/V232T) were also generated and studied.

### pH dependency of *Ef*Amy and site-directed variants.

The pH dependence of wild-type and mutant α-amylase activities were examined in various buffers at pH values that ranged from 5.0 to 11.0 under standard assay conditions. The results show that the optimal pH of all site-directed variants was 9, which was similar to that of the wild-type enzyme (see Fig. S2 in the supplemental material). While α-amylases from most bacteria and fungi have an optimal pH in the acidic to neutral range (1), *Ef*Amy represents an alkaline enzyme similar to the previously characterized alkaline α-amylases from alkalophilic Bacillus spp. (46). This can be considered an important property for utilizing the enzyme in detergent and textile industries and in other industrial applications.

### Effects of temperature on activity and stability of site-directed variants.

**FIG 4:**
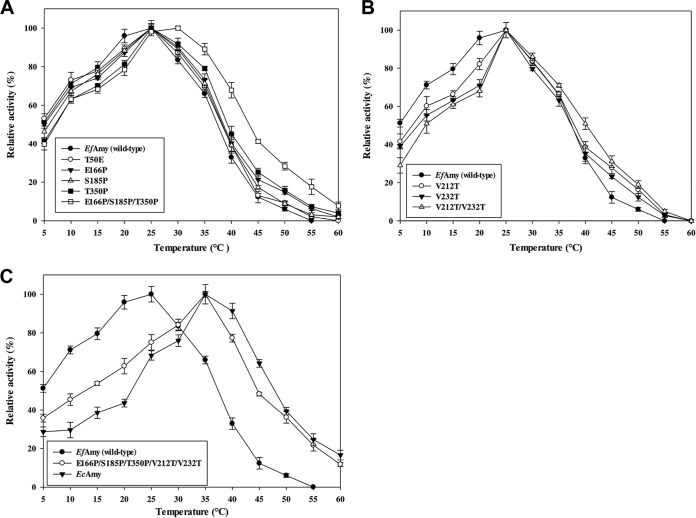
(A to C) Effect of temperature on the amylolytic activity of *Ef*Amy, *Ec*Amy, and mutants. For each enzyme, the total activity at the optimal temperature was set at 100%. This represented 1.27 U/ml for *Ef*Amy, 1.86 U/ml for *Ec*Amy, 1.31 U/ml for the E166P mutant, 1.34 U/ml for S185P, 1.31 U/ml for T350P, 1.53 U/ml for V212T, 1.51 U/ml for V232T, 1.91 U/ml for V212T/V232T, 1.32 U/ml for E166P/S185P/T350P, and 2.03 U/ml for E166P/S185P/T350P/V212T/V232T.

The effect of temperature on wild-type and mutant α-amylase activities was determined at various temperatures ranging from 5 to 60°C (Fig. 4). Under the standard conditions used, the wild-type *Ef*Amy showed the highest activity at approximately 25°C. Fifty percent maximal activity was observed at 5°C and approximately 10% residual activity remained at 45°C. All these characteristics indicate that the wild type behaves as a classic psychrophilic enzyme. To optimize the enzyme activity and stability at high temperatures and without a dramatic compromise of its activity at low temperatures for a broader application in industrial uses, we introduced mutations in both surface loops and in the catalytic domain of the *Ef*Amy based on our structural analysis. As shown in Fig. 4A and B, the mutant enzymes with single mutations (E166P, S185P, T350P, V212T, and V232T) showed optimal activity at 25°C, with only slightly decreased activity at low temperatures (5 to 20°C) and increased activity at high temperatures (30 to 55°C). These data may indicate that single mutagenesis is not sufficient to drastically modify the catalytic parameters of the cold-adapted enzyme to convert it to a true mesophilic α-amylase. Similar phenomena were also observed with a number of other microbial α-amylases (47–49). When the mutations were combined, the generated variant enzymes (E166P/S185P/T350P and E166P/S185P/T350P/V212T/V232T) displayed a shift in optimal temperature from 25 to 30°C that is typical of the mesophilic counterpart, *Ec*Amy (Fig. 4C). These results clearly demonstrate that the combination of mutations shows an effect of superimposing the activation that alters the behavior of the variant toward that of a mesophilic enzyme (50). As mentioned above, Pro has a more rigid conformation. Therefore, point mutations introducing extra Pro residues in the surface loops could thus increase the enzyme's surface rigidity. Recently, Brandsdal and coworkers disclosed the correlation between enzyme surface rigidity and temperature-dependent activity (51). Through computer simulation and mutation study, they determined that increasing the restraints in protein surface loops can lead to an unambiguous effect of turning a cold-adapted enzyme into a variant with mesophilic characteristics. On the other hand, our structural analysis indicates that the two mutations in the catalytic core region of the enzyme (V212T and V232T) may result in the formation of extra H bonds between the substituted residues and the catalytic sites (Fig. 3). It is known that the hydrogen bonding network around the catalytic domain of α-amylase can be crucial for the enzyme to maintain its stability and catalytic efficiency at a high temperature (52, 53), which is also supported by our results from this study.

**FIG 5:**
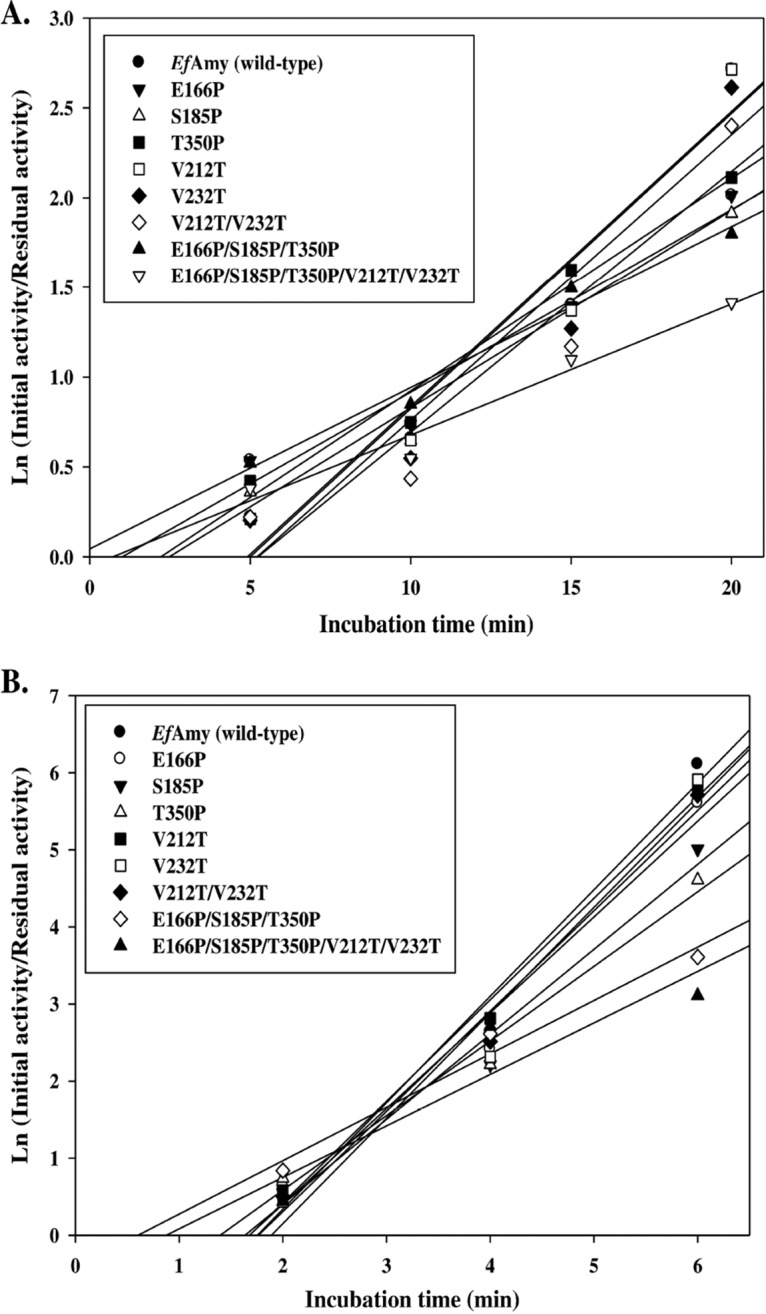
Thermostability of *Ef*Amy and mutants at 40°C (A) and 50°C (B).

To analyze the thermostability of the mutant α-amylases, we incubated the enzymes at 40 and 50°C for 2 to 20 min before measuring the residual activity at their optimal temperatures (Fig. 5). As shown in Table 1, all single mutant enzymes (E166P, S185P, T350P, V212T, and V232T) displayed increased stability at the same temperatures with respect to the wild-type *Ef*Amy, which showed 4.1- and 1.8-min half-lives at 40 and 50°C, respectively. In addition to single mutations, our study also examined the possible synergistic effects of combining mutations. Notably, the E166P/S185P/T350P/V212T/V232T variant, combining all the single mutations, was the most stable; half-life was more than 1.8-fold that of the wild-type enzyme at 50°C. Although single-site mutagenesis of the rationally selected residues was not able to raise the thermostability of the cold-adapted *Ef*Amy to the same level as *Ec*Amy, which represents the mesophilic counterpart, the half-lives are comparable when the combined mutations are taken into account (Table 1). These data reveal the effect of beneficial amino acid mutations that are synergistic or additive for the thermostability of the enzyme, which is consistent with the findings from other studies (54, 55). The generated variant enzyme is comparable to an engineered cold-adapted Pseudoalteromonas haloplanktis α-amylase that shows strong stabilization at room temperature (56). A number of previous studies have shown that both Pro insertions and hydrogen bonding play important roles in maintaining the thermostability of proteins (30, 57–59). In this study, by adopting sequence alignment and structural modeling analyses, we engineered *Ef*Amy with substituted Pro residues in the surface loops that promoted new hydrogen bonds in the catalytic domain. This most likely confers a reduced flexibility of the loops and catalytic core of *Ef*Amy and thus an increased thermostability of the enzyme.

**TABLE 1:** Stability properties of *Ef*Amy and site-directed variants

Enzyme or mutation(s)	*t*_1/2_ (min) at:	
	40°C	50°C
*Ef*Amy (wild type)	4.1	1.8
*Ec*Amy	6.5	4.2
E166P	5.0	2.4
S185P	5.2	2.6
T350P	4.8	2.3
V212T	4.2	2.0
V232T	4.3	2.0
V212T/V232T	4.6	2.2
E166P/S185P/T350P	5.3	3.0
E166P/S185P/T350P/V212T/V232T	5.7	3.3

### Kinetic parameters.

Kinetic studies were performed on the wild-type and mutated enzymes at 5, 25, and 35°C using starch as the substrate. As indicated by the results presented in Table 2, in comparison with the wild-type *Ef*Amy, the E166P, S185P, and T350P mutants showed slightly decreased turnover rates (*k*_cat_) and increased substrate binding affinities (decreased *K_m_*) at all temperatures. However, the overall catalytic efficiency was increased for these single-site mutants. This effect was greater when the three mutations were combined (E166P/S185P/T350P), with an increased *k*_cat_/*K_m_* value of 56% at 35°C. On the other hand, although slight decreases in *k*_cat_ values were observed at 5 and 25°C when the single-site variants with amino acid substitutions in the catalytic core of the enzyme (V212T or V232T) were tested, significant increases of catalytic efficiency were detected at all temperatures measured. This can be explained by the dramatic decrease in *K_m_* values for both single-site mutants. Interestingly, when the engineered enzyme that carried the combination of all mutations (E166P/S185P/T350P/V212T/V232T) was analyzed, a significant increase of turnover rate was observed at a high temperature (35°C). Indeed, this enzyme represents the most efficient catalyst among all the enzymes tested in this study when the reactions were carried out at 35°C. It is worth noting that the engineered enzyme that carried the five mutations combined showed an even better catalytic efficiency than the mesophilic *Ec*Amy (33). There is agreement that a trade-off between thermostability and catalytic activity has taken place during the natural evolution of enzymes to suit different temperature niches in the environment (60). This is supported by the fact that cold-adapted enzymes with high catalytic efficiency at a low temperature often show high thermolability at high temperatures due to the loss of native structures (61, 62). However, thermostability and catalytic activity are not mutually exclusive in a cold-adapted enzyme. It has been reported that directed-evolution methods can confer enzymes with both high thermostability and high catalytic activity (63, 64). D. Kern and coworkers have recently applied ancestral sequence reconstruction (ASR) approaches to create a “superenzyme” that displays high thermostability and catalytic activity at low temperatures (65). In this study, we further proved that rational design-based protein engineering can also achieve the same effect.

**TABLE 2:** Kinetic parameters of *Ef*Amy and site-directed variants at 5, 25, and 35°C

Enzyme or mutation(s)	Temp (°C)	*k*_cat_ (s^−1^)	*K_m_* (g · liter^−1^)	*k*_cat_/*K_m_* (s^−1^ · g^−1^ · liter)
*Ef*Amy	5	718.55 ± 31.91	3.05 ± 0.08	235.59 ± 16.65
	25	1466.42 ± 46.1	3.31 ± 0.16	443.03 ± 35.43
	35	982.5 ± 33.18	4.43 ± 0.19	221.78 ± 17.03
*Ec*Amy	5	377.53 ± 19.33	1.31 ± 0.04	288.19 ± 23.58
	25	618.91 ± 23.92	1.36 ± 0.03	455.08 ± 27.64
	35	879.76 ± 27.61	1.42 ± 0.07	619.55 ± 50.11
E166P	5	694.31 ± 26.29	2.91 ± 0.06	238.59 ± 13.96
	25	1452.39 ± 37.49	3.21 ± 0.07	452.46 ± 21.56
	35	1033,78 ± 38.91	4.01 ± 0.12	262.79 ± 17.58
S185P	5	677.62 ± 26.41	2.86 ± 0.13	236.93 ± 20.05
	25	1407.58 ± 33.96	3.04 ± 0.11	463.02 ± 27.96
	35	1162.17 ± 31.42	3.89 ± 0.08	298.76 ± 14.23
T350P	5	704.62 ± 23.19	2.99 ± 0.09	235.65 ± 14.86
	25	1458.29 ± 32.48	3.23 ± 0.08	451.48 ± 21.25
	35	999.76 ± 29.35	4.18 ± 0.18	239.18 ± 17.35
V212T	5	681.49 ± 28.67	2.36 ± 0.07	288.77 ± 20.73
	25	1392.11 ± 42.49	2.67 ± 0.11	521.39 ± 37.46
	35	1002.34 ± 34.72	2.81 ± 0.16	356.71 ± 32.77
V232T	5	693.89 ± 27.33	2.42 ± 0.15	286.73 ± 29.18
	25	1409.63 ± 37.54	2.66 ± 0.09	529.94 ± 32.08
	35	1011.87 ± 39.69	2.81 ± 0.12	360.1 ± 29.56
V212T/V232T	5	643.28 ± 31.42	1.76 ± 0.13	365.51 ± 45.1
	25	1314.08 ± 41.66	1.89 ± 0.09	695.28 ± 55.28
	35	1039.36 ± 34.51	2.01 ± 0.11	517.09 ± 45.61
E166P/S185P/T350P	5	632.57 ± 21.65	2.69 ± 0.11	235.16 ± 17.69
	25	1297.12 ± 41.6	2.89 ± 0.13	448.83 ± 34.65
	35	1304 ± 38.85	3.76 ± 0.18	346.81 ± 27.01
E166P/S185P/T350P/V212T/V232T	5	483.75 ± 23.28	1.53 ± 0.05	316.18 ± 25.58
	25	983.16 ± 29.61	1.59 ± 0.07	618.34 ± 45.93
	35	1332.03 ± 38.37	1.67 ± 0.11	797.6 ± 75.84

### Conclusion.

Protein engineering strategies are often used to optimize enzyme traits. Both rational design and directed evolution have been used to tailor enzyme properties (66, 67). Rational design requires a thorough understanding of parental structures, consensus sequences, and interactions between amino acid residues to identify mutations that will lead to desired enzyme properties, especially the high level of thermostability and catalytic efficiency required for industrial applications (68). In this work, we applied a rational design method to engineer a novel cold-adapted α-amylase with improved thermostability, substrate binding affinity, and catalytic efficiency to various extents. In one variant (E166P/S185P/T350P), we introduced Pro residues in the A and B domains of surface loops. In the second variant (V212T/V232T), we facilitated extra molecular interactions in the catalytic core of the protein. The aim of these substitutions was to rigidify the molecular structure of the enzyme. Indeed, the E166P/S185P/T350P site-directed variant exhibited a strong increase in thermostability in comparison to that of the wild type, whereas the V212T/V232T site-directed mutation had a high impact on the catalytic efficiency compared with those of the enzymes with surface mutations. All our mutation sites were selected based on structural and consensus analyses of the cold-adapted enzyme and its mesophilic counterpart. Unlike the site-directed mutagenesis solely based on protein structural analysis that often shows adverse impacts on the kinetic parameters, the structure-guided consensus approach assumes that nature has efficiently optimized the protein sequence space. Consequently, the targeting of a single mutational point of interest at positions which match the desired consensus cutoff has proven to be a successful approach for engineering enzymes with improved kinetic parameters (30, 69, 70). The combination of sequence analysis and homology-based model comparison appears to be a good approach for the rational design of mutations to improve the thermal stability and catalytic efficiency of this enzyme (71, 72). Our study supports this multidisciplinary approach for engendering structural diversities in catalysis and for optimizing particular industrial applications. The E. focardii α-amylase that we have engineered is promising for industrial applications not only for its efficiency in the cold but also because it represents an alkaline enzyme, similar to the α-amylase from alkalophilic Bacillus spp. (11, 46) and different from most of the α-amylases characterized so far. These characteristics can be considered important properties for use in detergent and textile industries and in other industrial applications.

## MATERIALS AND METHODS

### Materials.

Restriction enzymes, recombinant *Taq* DNA polymerase, and *Pfu* DNA polymerase were purchased from Fermentas (Milan, Italy). Ampicillin, chloramphenicol, isopropyl-β-d-thiogalactopyranoside (IPTG), 5-bromo-4-chloro-indolyl-β-d-galactopyranoside (X-Gal), and soluble starch were purchased from Sigma-Aldrich (USA). All chemicals were reagent grade. All oligonucleotide primers used in this study were synthesized by Sigma (Milan, Italy).

### Bacterial strains, plasmids, and culture conditions.

E. coli DH5α was used as the host for cloning, whereas E. coli BL21(DE3)/pLysS harbored the wild-type and recombinant plasmids for gene expression. The pET22b(+) vector (Novagen), containing the inducible T7 promoter, was used as the expression vector. The plasmid pET22b-*EfAmy*, containing the gene encoding *Ef*Amy (33), was used for production of the wild-type *Ef*Amy protein. Different E. coli strains harboring wild-type and mutated genes were routinely grown in Luria-Bertani (LB) medium at 37°C. When required, antibiotics and chromogenic substrates were added at the following concentrations: 100 μg/ml ampicillin, 34 μg/ml chloramphenicol, and 30 μg/ml X-Gal.

### Construction, expression, and purification of the mutant plasmids.

The gene encoding the wild-type *Ef*Amy was previously cloned and overexpressed in E. coli BL21(DE3)/pLysS (28), while the mutated genes encoding the mutated *Ef*Amy were constructed by PCR-based site-directed mutagenesis (73). For each single mutation, PCRs were carried out using the plasmid pET-*Ef*Amy as the template, *Pfu* DNA polymerase, and two complementary mutagenic primers for each site-specific mutation. For each combined mutation, PCR was performed using the former mutated plasmid as the template with the corresponding primers for each reaction. The sequences of the oligonucleotides are shown in Table S1 in the supplemental material. The final amplified products were verified by bidirectional DNA sequencing. As a result, PCR mutagenesis yielded eight expression plasmids: pET-*EfAmy*-E166P, pET-*EfAmy*-S185P, pET-*EfAmy*-T350P, pET-*EfAmy*-V212T, pET-*EfAmy*-V232T, pET-*EfAmy*-V212T/V232T, pET-*EfAmy*-E166P/S185P/T350P, and pET-*EfAmy*-E166P/S185P/T350P/V212T/V232T, where the numbers indicate the position in the polypeptide of the mutated residue and the letters before and after the numbers indicate the original and the substituted amino acid, respectively.

E. coli BL21(DE3)/pLysS cells carrying wild-type and mutant *Ef*Amy plasmids were grown overnight at 37°C in LB medium supplemented with 100 μg/ml ampicillin and 34 μg/ml chloramphenicol. The overnight cultures were diluted to an optical density at 600 nm (OD_600_) of approximately 0.08 in 600 ml of LB medium supplemented with 100 μg/ml ampicillin and 34 μg/ml chloramphenicol using a 2-liter flask. Cultivation took place in a 2-liter flask at 30°C under vigorous stirring and aeration. The induction procedures were carried out when cultures reached an OD_600_ of 0.6 to 0.8 by the addition of filter-sterilized IPTG to a final concentration of 0.1 mM. The cultures were grown overnight for postinduction. Cells were harvested by centrifugation at 5,000 × *g* and 4°C for 20 min, were divided into 0.50-g aliquots, and were frozen at −80°C.

The IPTG induction of recombinant E. coli BL21(DE3)/pLysS cells resulted in the accumulation of recombinant E. focardii α-amylases as inclusion bodies. The recovery procedure was conducted according to a previous description (33).

SDS-PAGE (with 12% polyacrylamide) was performed by the method of Laemmli (25). The protein concentration was determined according to the Bradford method with bovine serum albumin as the standard (74). As estimated from SDS-PAGE (see Fig. S1), the molecular masses of the purified proteins were between 52 and 54 kDa.

### Enzyme assays.

The α-amylase enzyme activity was measured according to the modified method described by Xiao et al. (75). The reactions for both the wild-type and mutant enzymes were carried out by adding 0.1 ml of starch solution (at a concentration of 2 g/liter) as a substrate to 0.1 ml of a solution containing α-amylases (wild-type or mutated) at a concentration of 0.4 mg/ml in 0.1 M Tris-HCl buffer (pH 9). All the reaction conditions are described in detail in the following paragraphs. The α-amylase activity was confirmed by adding 0.1 ml of iodine reagent (0.5% KI and 0.05% I_2_) to the solution. Following color development, the formation of the starch-iodine complex was monitored on the spectrophotometer at 580 nm (A_580_). One unit of α-amylase activity is defined as the disappearance of an average of 1 mg of iodine-binding starch material per min in the assay reaction.

### Effect of pH on enzyme activity.

The optimal pH of the wild-type and mutant α-amylases was determined by incubating the assay reaction mixtures for 20 min at 25°C in the following buffers (all at a concentration of 0.1 M): morpholineethanesulfonic acid (MES; pH range, 5.0 to 7.0), Tris-HCl (pH range, 7.0 to 9.0), and glycine-NaOH (pH range, 9.0 to 11.0).

### Effect of temperature on enzyme activity and stability.

The optimal temperatures of wild-type and mutant α-amylases were determined by incubating the reactions at temperatures ranging from 0 to 60°C in 0.1 M Tris-HCl buffer (pH 9). Half-lives of thermal inactivation were determined for purified α-amylases by incubating the enzymes in 0.1 M Tris-HCl buffer (pH 9) at 40°C and 50°C for 0 to 20 min at regular time intervals. Initial and residual activities were measured under the standard assay conditions as described above. The first-order rate constant, *K_d_*, of irreversible thermal denaturation was obtained from the slope of the plots of ln (initial activity/residual activity) versus time, and the half-lives (*t*_1/2_) were calculated as ln 2/*K_d_*.

### Influence of mutations on kinetic parameters.

The kinetic parameters *K_m_* and *k*_cat_ of wild-type and mutant α-amylases were measured using soluble starch as the substrate at 5, 25, and 35°C. The initial velocities of substrate hydrolysis were monitored for a minimum of substrate concentration. The substrate concentrations used ranged from 0.5 to 8 g/liter. All kinetic data were analyzed by nonlinear regression using Origin 8.0. The standard errors for each parameter were estimated from the curve fitting. Assays were performed in duplicate, and results for kinetic data were the means from two independent experiments.

### Sequence analysis, phylogenetic tree construction, and comparative modeling.

Sequence similarity and analysis for conserved sequence regions (CSR) were performed using BLAST programs on the National Center for Biotechnology Information (NCBI) website (http://www.ncbi.nlm.nih.gov). Sequence alignment was performed using Clustal Omega (http://www.ebi.ac.uk/Tools/msa/clustalo/), and the related phylogenetic tree was calculated using the neighbor-joining method and displayed using the R package APE (version 3.5) (76). The comparative homology models of E. focardii and E. crassus α-amylases were obtained by the Modeler software (77) using TAKA α-amylase A (TAA) from Aspergillus oryzae (PDB no. 2TAA and 2GVY) (78, 79) and the α-amylase from Malbranchea cinnamomea (PDB no. 3VM7) (80) as specific templates. The sequence identities shown by these three templates against *Ef*Amy were 33.3%, 33.8% and 32.9%, respectively, and against *Ec*Amy the identities were 37.4%, 37.6%, and 35.9%, respectively. It is relevant to note that each template had a coverage percentage higher than 95%. To visualize residues involved in ligand interaction, the *Ef*Amy homology model was superimposed on the structure of TAA from A. oryzae in complex with ABC, an acarbose-derived hexasaccharide, as the ligand (PDB no. 7TAA) (43). The Deep View Swiss-PDB viewer software and PyMOL v1.5 were used to visualize and analyze the three-dimensional models.

## Supplementary Material

Supplemental material

## References

[1] Pandey A, Nigam P, Soccol CR, Soccol VT, Singh D, Mohan R 2000 Advances in microbial amylases. Biotechnol Appl Biochem 31(Pt 2):135–152. 10744959 10.1042/ba19990073

[2] van der Maarel MJ, van Der Veen B, Uitdehaag JC, Leemhuis H, Dijkhuizen L 2002 Properties and applications of starch-converting enzymes of the α-amylase family. J Biotechnol 94:137–155. doi:10.1016/S0168-1656(01)00407-2. 11796168

[3] Janeček Š, Svensson B, MacGregor EA 2014 α-Amylase: an enzyme specificity found in various families of glycoside hydrolases. Cell Mol Life Sci 71:1149–1170. doi:10.1007/s00018-013-1388-z. 23807207 PMC11114072

[4] Lombard V, Golaconda Ramulu H, Drula E, Coutinho PM, Henrissat B 2014 The carbohydrate-active enzymes database (CAZy) in 2013. Nucleic Acids Res 42:D490–D495. doi:10.1093/nar/gkt1178. 24270786 PMC3965031

[5] Janeček Š, Gabriško M 2016 Remarkable evolutionary relatedness among the enzymes and proteins from the α-amylase family. Cell Mol Life Sci 73:2707–2725. doi:10.1007/s00018-016-2246-6. 27154042 PMC11108405

[6] MacGregor EA, Janeček Š, Svensson B 2001 Relationship of sequence and structure to specificity in the α-amylase family of enzymes. Biochim Biophys Acta 1546:1–20. doi:10.1016/S0167-4838(00)00302-2. 11257505

[7] Nielsen JE, Borchert TV 2000 Protein engineering of bacterial α-amylases. Biochim Biophys Acta 1543:253–274. doi:10.1016/S0167-4838(00)00240-5. 11150610

[8] Khemakhem B, Ali MB, Aghajari N, Juy M, Haser R, Bejar S 2009 Engineering of the α-amylase from *Geobacillus stearothermophilus* US100 for detergent incorporation. Biotechnol Bioeng 102:380–389. doi:10.1002/bit.22083. 18951544

[9] Gupta R, Gigras P, Mohapatra H, Goswami VK, Chauhan B 2003 Microbial *α*-amylases: a biotechnological perspective. Process Biochem 38:1599–1616. doi:10.1016/S0032-9592(03)00053-0.

[10] Saito N 1973 A thermophilic extracellular α-amylase from *Bacillus licheniformis*. Arch Biochem Biophys 155:290–298. doi:10.1016/0003-9861(73)90117-3. 4705426

[11] Burhan A, Nisa U, Gökhan C, Ömer C, Ashabil A, Osman G 2003 Enzymatic properties of a novel thermostable, thermophilic, alkaline and chelator resistant amylase from an alkaliphilic *Bacillus* sp. isolate ANT-6. Process Biochem 38:1397–1403. doi:10.1016/S0032-9592(03)00037-2.

[12] Khajeh K, Nemat-Gorgani M 2001 Comparative studies on a mesophilic and a thermophilic α-amylase. Appl Biochem Biotechnol 90:47–55. doi:10.1385/ABAB:90:1:47. 11257806

[13] Janeček Š, Baláž Š 1992 α-Amylases and approaches leading to their enhanced stability. FEBS Lett 304:1–3. doi:10.1016/0014-5793(92)80575-2. 1618293

[14] Samie N, Noghabi KA, Gharegozloo Z, Zahiri HS, Ahmadian G, Sharafi H, Behrozi R, Vali H 2012 Psychrophilic α-amylase from *Aeromonas veronii* NS07 isolated from farm soils. Process Biochem 47:1381–1387. doi:10.1016/j.procbio.2012.05.007.

[15] Feller G, Narinx E, Arpigny JL, Aittaleb M, Baise E, Genicot S, Gerday C 1996 Enzymes from psychrophilic organisms. FEMS Microbiol Rev 18:189–202. doi:10.1111/j.1574-6976.1996.tb00236.x.

[16] Cavicchioli R, Charlton T, Ertan H, Omar SM, Siddiqui K, Williams T 2011 Biotechnological uses of enzymes from psychrophiles. Microb Biotechnol 4:449–460. doi:10.1111/j.1751-7915.2011.00258.x. 21733127 PMC3815257

[17] Gerday C, Aittaleb M, Bentahir M, Chessa J-P, Claverie P, Collins T, D'Amico S, Dumont J, Garsoux G, Georlette D 2000 Cold-adapted enzymes: from fundamentals to biotechnology. Trends Biotechnol 18:103–107. doi:10.1016/S0167-7799(99)01413-4. 10675897

[18] Zhang J-W, Zeng R-Y 2008 Purification and characterization of a cold-adapted α-amylase produced by *Nocardiopsis* sp. 7326 isolated from Prydz Bay, Antarctic. Mar Biotechnol (NY) 10:75–82. doi:10.1007/s10126-007-9035-z. 17934774

[19] Aghajari N, Haser R, Feller G, Gerday C 1996 Crystallization and preliminary X-ray diffraction studies of α-amylase from the Antarctic psychrophile *Alteromonas haloplanctis* A23. Protein Sci 5:2128–2129. doi:10.1002/pro.5560051021. 8897615 PMC2143274

[20] Morita Y, Nakamura T, Hasan Q, Murakami Y, Yokoyama K, Tamiya E 1997 Cold-active enzymes from cold-adapted bacteria. J Am Oil Chem Soc 74:441–444. doi:10.1007/s11746-997-0103-3.

[21] Aghajari N, Feller G, Gerday C, Haser R 1998 Structures of the psychrophilic *Alteromonas haloplanctis* α-amylase give insights into cold adaptation at a molecular level. Structure 6:1503–1516. doi:10.1016/S0969-2126(98)00149-X. 9862804

[22] D'Amico S, Gerday C, Feller G 2001 Structural determinants of cold adaptation and stability in a large protein. J Biol Chem 276:25791–25796. doi:10.1074/jbc.M102741200. 11325973

[23] Cavicchioli R, Siddiqui KS, Andrews D, Sowers KR 2002 Low-temperature extremophiles and their applications. Curr Opin Biotechnol 13:253–261. doi:10.1016/S0958-1669(02)00317-8. 12180102

[24] Groudieva T, Kambourova M, Yusef H, Royter M, Grote R, Trinks H, Antranikian G 2004 Diversity and cold-active hydrolytic enzymes of culturable bacteria associated with Arctic sea ice, Spitzbergen. Extremophiles 8:475–488. doi:10.1007/s00792-004-0409-0. 15252724

[25] Laemmli UK 1970 Cleavage of structural proteins during the assembly of the head of bacteriophage T4. Nature 227:680–685. 5432063 10.1038/227680a0

[26] Place SP, Hofmann GE 2005 Constitutive expression of a stress-inducible heat shock protein gene, hsp70, in phylogenetically distant Antarctic fish. Polar Biol 28:261–267. doi:10.1007/s00300-004-0697-y.

[27] Margesin R 2009 Cold-active enzymes as new tools in biotechnology, p 164–184. *In* Gerday C, Glansdorff N (ed), Extremophiles–volume II. Eolss Publishers, Paris, France.

[28] Lehmann M, Wyss M 2001 Engineering proteins for thermostability: the use of sequence alignments versus rational design and directed evolution. Curr Opin Biotechnol 12:371–375. doi:10.1016/S0958-1669(00)00229-9. 11551465

[29] Mabrouk SB, Aghajari N, Ali MB, Messaoud EB, Juy M, Haser R, Bejar S 2011 Enhancement of the thermostability of the maltogenic amylase MAUS149 by Gly312Ala and Lys436Arg substitutions. Bioresour Technol 102:1740–1746. doi:10.1016/j.biortech.2010.08.082. 20855205

[30] Duan X, Chen J, Wu J 2013 Improving the thermostability and catalytic efficiency of *Bacillus deramificans* pullulanase by site-directed mutagenesis. Appl Environ Microbiol 79:4072–4077. doi:10.1128/AEM.00457-13. 23624477 PMC3697558

[31] Yang G, De Santi C, de Pascale D, Pucciarelli S, Pucciarelli S, Miceli C 2013 Characterization of the first eukaryotic cold-adapted patatin-like phospholipase from the psychrophilic *Euplotes focardii*: identification of putative determinants of thermal-adaptation by comparison with the homologous protein from the mesophilic *Euplotes crassus*. Biochimie 95:1795–1806. doi:10.1016/j.biochi.2013.06.008. 23796575

[32] van den Burg B, Eijsink VG 2002 Selection of mutations for increased protein stability. Curr Opin Biotechnol 13:333–337. doi:10.1016/S0958-1669(02)00325-7. 12323355

[33] Yang G, Yang G, Aprile L, Turturo V, Pucciarelli S, Pucciarelli S, Miceli C 2013 Characterization and comparative analysis of psychrophilic and mesophilic alpha-amylases from *Euplotes* species: a contribution to the understanding of enzyme thermal adaptation. Biochem Biophys Res Commun 438:715–720. doi:10.1016/j.bbrc.2013.07.113. 23916704

[34] Chiappori F, Pucciarelli S, Merelli I, Ballarini P, Miceli C, Milanesi L 2012 Structural thermal adaptation of β-tubulins from the Antarctic psychrophilic protozoan *Euplotes focardii*. Proteins 80:1154–1166. doi:10.1002/prot.24016. 22275059

[35] Pucciarelli S, La Terza A, Ballarini P, Barchetta S, Yu T, Marziale F, Passini V, Methé B, Detrich HW, Miceli C 2009 Molecular cold-adaptation of protein function and gene regulation: the case for comparative genomic analyses in marine ciliated protozoa. Mar Genomics 2:57–66. doi:10.1016/j.margen.2009.03.008. 21798173

[36] Sarmiento F, Peralta R, Blamey JM 2015 Cold and hot extremozymes: industrial relevance and current trends. Front Bioeng Biotechnol 3:148. doi:10.3389/fbioe.2015.00148. 26539430 PMC4611823

[37] Brady R, Brzozowski A, Derewenda Z, Dodson E, Dodson G 1991 Solution of the structure of *Aspergillus niger* acid α-amylase by combined molecular replacement and multiple isomorphous replacement methods. Acta Crystallogr B 47:527–535. doi:10.1107/S0108768191001908. 1930834

[38] Tada S, Iimura Y, Gomi K, Takahashi K, Hara S, Yoshizawa K 1989 Cloning and nucleotide sequence of the genomic Taka-amylase A gene of *Aspergillus oryzae*. Agric Biol Chem 53:593–599. doi:10.1271/bbb1961.53.593.

[39] Itoh T, Yamashita I, Fukui S 1987 Nucleotide sequence of the α-amylase gene (ALP1) in the yeast *Saccharomycopsis fibuligera*. FEBS Lett 219:339–342. doi:10.1016/0014-5793(87)80248-X. 3497057

[40] Chen W, Xie T, Shao Y, Chen F 2012 Phylogenomic relationships between amylolytic enzymes from 85 strains of fungi. PLoS One 7:e49679. doi:10.1371/journal.pone.0049679. 23166747 PMC3499471

[41] Lo H-F, Lin L-L, Chiang W-Y, Chie M-C, Hsu W-H, Chang C-T 2002 Deletion analysis of the C-terminal region of the α-amylase of *Bacillus* sp. strain TS-23. Arch Microbiol 178:115–123. doi:10.1007/s00203-002-0431-5. 12115056

[42] Feller G, Gerday C 1997 Psychrophilic enzymes: molecular basis of cold adaptation. Cell Mol Life Sci 53:830–841. doi:10.1007/s000180050103. 9413552 PMC11147173

[43] Brzozowski AM, Davies GJ 1997 Structure of the *Aspergillus oryzae* alpha-amylase complexed with the inhibitor acarbose at 2.0 A resolution. Biochemistry 36:10837–10845. doi:10.1021/bi970539i. 9283074

[44] Jaouadi B, Aghajari N, Haser R, Bejar S 2010 Enhancement of the thermostability and the catalytic efficiency of *Bacillus pumilus* CBS protease by site-directed mutagenesis. Biochimie 92:360–369. doi:10.1016/j.biochi.2010.01.008. 20096326

[45] Ben-Naim A 2013 Theory of cold denaturation of proteins. Adv Biol Chem 3:29–39. doi:10.4236/abc.2013.31005.

[46] Lee S-P, Morikawa M, Takagi M, Imanaka T 1994 Cloning of the aapT gene and characterization of its product, alpha-amylase-pullulanase (AapT), from thermophilic and alkaliphilic *Bacillus* sp. strain XAL601. Appl Environ Microbiol 60:3764–3773. 7986049 10.1128/aem.60.10.3764-3773.1994 PMC201885

[47] Igarashi K, Hatada Y, Ikawa K, Araki H, Ozawa T, Kobayashi T, Ozaki K, Ito S 1998 Improved thermostability of a *Bacillus* α-amylase by deletion of an arginine-glycine residue is caused by enhanced calcium binding. Biochem Biophys Res Commun 248:372–377. doi:10.1006/bbrc.1998.8970. 9675143

[48] Bisgaard-Frantzen H, Svendsen A, Norman B, Pedersen S, Kjaerulff S, Outtrup H, Borchert TV 1999 Development of industrially important α-amylases. J Appl Glycosci 46:199–206. doi:10.5458/jag.46.199.

[49] Chi M-C, Chen Y-H, Wu T-J, Lo H-F, Lin L-L 2010 Engineering of a truncated α-amylase of *Bacillus* sp. strain TS-23 for the simultaneous improvement of thermal and oxidative stabilities. J Biosci Bioeng 109:531–538. doi:10.1016/j.jbiosc.2009.11.012. 20471589

[50] D'Amico S, Marx J-C, Gerday C, Feller G 2003 Activity-stability relationships in extremophilic enzymes. J Biol Chem 278:7891–7896. doi:10.1074/jbc.M212508200. 12511577

[51] Isaksen GV, Åqvist J, Brandsdal BO 2016 Enzyme surface rigidity tunes the temperature dependence of catalytic rates. Proc Natl Acad Sci U S A 133:7822–7827. doi:10.1073/pnas.1605237113. PMC4948340 27354533

[52] Liu Y-H, Lu F-P, Li Y, Wang J-L, Gao C 2008 Acid stabilization of *Bacillus licheniformis* alpha amylase through introduction of mutations. Appl Microbiol Biotechnol 80:795–803. doi:10.1007/s00253-008-1580-5. 18626642

[53] Yang H, Liu L, Shin H-D, Chen RR, Li J, Du G, Chen J 2013 Structure-based engineering of histidine residues in the catalytic domain of α-amylase from Bacillus subtilis for improved protein stability and catalytic efficiency under acidic conditions. J Biotechnol 164:59–66. doi:10.1016/j.jbiotec.2012.12.007. 23262127

[54] Liu L, Deng Z, Yang H, Li J, Shin H-D, Chen RR, Du G, Chen J 2014 In silico rational design and systems engineering of disulfide bridges in the catalytic domain of an alkaline α-amylase from *Alkalimonas amylolytica* to improve thermostability. Appl Environ Microbiol 80:798–807. doi:10.1128/AEM.03045-13. 24212581 PMC3911192

[55] Voutilainen SP, Murray PG, Tuohy MG, Koivula A 2010 Expression of *Talaromyces emersonii* cellobiohydrolase Cel7A in *Saccharomyces cerevisiae* and rational mutagenesis to improve its thermostability and activity. Protein Eng Des Sel 23:69–79. doi:10.1093/protein/gzp072. 19951999

[56] D'Amico S, Gerday C, Feller G 2003 Temperature adaptation of proteins: engineering mesophilic-like activity and stability in a cold-adapted α-amylase. J Mol Biol 332:981–988. doi:10.1016/j.jmb.2003.07.014. 14499602

[57] Muslin E, Clark S, Henson C 2002 The effect of proline insertions on the thermostability of a barley α-glucosidase. Protein Eng 15:29–33. doi:10.1093/protein/15.1.29. 11842235

[58] Zhou C, Xue Y, Ma Y 2010 Enhancing the thermostability of α-glucosidase from *Thermoanaerobacter tengcongensis* MB4 by single proline substitution. J Biosci Bioeng 110:12–17. doi:10.1016/j.jbiosc.2009.12.002. 20541109

[59] Vogt G, Woell S, Argos P 1997 Protein thermal stability, hydrogen bonds, and ion pairs. J Mol Biol 269:631–643. doi:10.1006/jmbi.1997.1042. 9217266

[60] Siddiqui KS, Cavicchioli R 2006 Cold-adapted enzymes. Annu Rev Biochem 75:403–433. doi:10.1146/annurev.biochem.75.103004.142723. 16756497

[61] Arnold FH, Wintrode PL, Miyazaki K, Gershenson A 2001 How enzymes adapt: lessons from directed evolution. Trends Biochem Sci 26:100–106. doi:10.1016/S0968-0004(00)01755-2. 11166567

[62] Feller G, Gerday C 2003 Psychrophilic enzymes: hot topics in cold adaptation. Nat Rev Microbiol 1:200–208. doi:10.1038/nrmicro773. 15035024

[63] Merz A, Yee M, Szadkowski H, Pappenberger G, Crameri A, Stemmer WP, Yanofsky C, Kirschner K 2000 Improving the catalytic activity of a thermophilic enzyme at low temperatures. Biochemistry 39:880–889. doi:10.1021/bi992333i. 10653631

[64] Wintrode PL, Miyazaki K, Arnold FH 2000 Cold adaptation of a mesophilic subtilisin-like protease by laboratory evolution. J Biol Chem 275:31635–31640. doi:10.1074/jbc.M004503200. 10906329

[65] Nguyen V, Wilson C, Hoemberger M, Stiller JB, Agafonov RV, Kutter S, English J, Theobald DL, Kern D 2017 Evolutionary drivers of thermoadaptation in enzyme catalysis. Science 355:289–294. doi:10.1126/science.aah3717. 28008087 PMC5649376

[66] Toscano MD, Woycechowsky KJ, Hilvert D 2007 Minimalist active-site redesign: teaching old enzymes new tricks. Angew Chem Int Ed Engl 46:3212–3236. doi:10.1002/anie.200604205. 17450624

[67] Penning TM, Jez JM 2001 Enzyme redesign. Chem Rev 101:3027–3046. doi:10.1021/cr000049n. 11710061

[68] Yang G, Ding Y 2014 Recent advances in biocatalyst discovery, development and applications. Bioorg Med Chem 22:5604–5612. doi:10.1016/j.bmc.2014.06.033. 25042559

[69] Dror A, Shemesh E, Dayan N, Fishman A 2014 Protein engineering by random mutagenesis and structure-guided consensus of *Geobacillus stearothermophilus* lipase T6 for enhanced stability in methanol. Appl Environ Microbiol 80:1515–1527. doi:10.1128/AEM.03371-13. 24362426 PMC3911059

[70] Bommarius AS, Blum JK, Abrahamson MJ 2011 Status of protein engineering for biocatalysts: how to design an industrially useful biocatalyst. Curr Opin Chem Biol 15:194–200. doi:10.1016/j.cbpa.2010.11.011. 21115265

[71] Bogin O, Peretz M, Hacham Y, Burstein Y, Korkhin Y, Frolow F 1998 Enhanced thermal stability of *Clostridium beijerinckii* alcohol dehydrogenase after strategic substitution of amino acid residues with prolines from the homologous thermophilic *Thermoanaerobacter brockii* alcohol dehydrogenase. Protein Sci 7:1156–1163. doi:10.1002/pro.5560070509. 9836874 PMC2144005

[72] Tsigos I, Mavromatis K, Tzanodaskalaki M, Pozidis C, Kokkinidis M, Bouriotis V 2001 Engineering the properties of a cold active enzyme through rational redesign of the active site. Eur J Biochem 268:5074–5080. doi:10.1046/j.0014-2956.2001.02432.x. 11589698

[73] Ho SN, Hunt HD, Horton RM, Pullen JK, Pease LR 1989 Site-directed mutagenesis by overlap extension using the polymerase chain reaction. Gene 77:51–59. doi:10.1016/0378-1119(89)90358-2. 2744487

[74] Kruger NJ 1994 The Bradford method for protein quantitation. Methods Mol Biol 32:9–15. doi:10.1385/0-89603-268-X:9. 7951753

[75] Xiao Z, Storms R, Tsang A 2006 A quantitative starch-iodine method for measuring alpha-amylase and glucoamylase activities. Anal Biochem 351:146–148. doi:10.1016/j.ab.2006.01.036. 16500607

[76] Paradis E, Claude J, Strimmer K 2004 APE: analyses of phylogenetics and evolution in R language. Bioinformatics 20:289–290. doi:10.1093/bioinformatics/btg412. 14734327

[77] Sali A, Blundell TL 1993 Comparative protein modelling by satisfaction of spatial restraints. J Mol Biol 234:779–815. doi:10.1006/jmbi.1993.1626. 8254673

[78] Matsuura Y, Kusunoki M, Harada W, Kakudo M 1984 Structure and possible catalytic residues of Taka-amylase A. J Biochem 95:697–702. 6609921 10.1093/oxfordjournals.jbchem.a134659

[79] Vujicic-Zagar A, Dijkstra BW 2006 Monoclinic crystal form of Aspergillus niger alpha-amylase in complex with maltose at 1.8 angstroms resolution. Acta Crystallogr Sect F Struct Biol Cryst Commun 62:716–721. doi:10.1107/S1744309106024729. PMC2242925 16880540

[80] Han P, Zhou P, Hu S, Yang S, Yan Q, Jiang Z 2013 A novel multifunctional alpha-amylase from the thermophilic fungus *Malbranchea cinnamomea*: biochemical characterization and three-dimensional structure. Appl Biochem Biotechnol 170:420–435. doi:10.1007/s12010-013-0198-y. 23536251

[81] van der Kaaij R, Janeček Š, van der Maarel M, Dijkhuizen L 2007 Phylogenetic and biochemical characterization of a novel cluster of intracellular fungal α-amylase enzymes. Microbiology 153:4003–4015. doi:10.1099/mic.0.2007/008607-0. 18048915

[82] Machius M, Wiegand G, Huber R 1995 Crystal structure of calcium-depleted *Bacillus licheniformis* α-amylase at 2.2 Å resolution. J Mol Biol 246:545–559. doi:10.1006/jmbi.1994.0106. 7877175

