# Supplementary material for: Rational Engineering of a Cold-Adapted α-Amylase from the Antarctic Ciliate Euplotes focardii for Simultaneous Improvement of Thermostability and Catalytic Activity
Source: Appl Environ Microbiol. 2017 Jun 16;83(13):e00449-17. doi: 10.1128/AEM.00449-17 (PMC5478988; doi:10.1128/AEM.00449-17)
Supplement: Supplemental material [file supp_83_13_e00449-17__index.html]

Supplemental material 

# Rational Engineering of a Cold-Adapted α-Amylase from the Antarctic Ciliate Euplotes focardii for Simultaneous Improvement of Thermostability and Catalytic Activity

## Supplemental material

- Supplemental file 1 -

  Nucleotide sequences of primers used for site-directed mutagenesis (Table S1); GH13 subfamily enzymes used in the phylogenetic analysis (Table S2); SDS-PAGE of recombinant α-amylases (Fig. S1); effect of pH on the amylolytic activity of *Ef*Amy and mutants (Fig. S2).

  PDF, 127K
