# Supplementary material for: Rational Engineering of a Cold-Adapted α-Amylase from the Antarctic Ciliate Euplotes focardii for Simultaneous Improvement of Thermostability and Catalytic Activity
Source: Appl Environ Microbiol. 2017 Jun 16;83(13):e00449-17. doi: 10.1128/AEM.00449-17 (PMC5478988; doi:10.1128/AEM.00449-17)
Supplement: Supplemental material [file AEM.00449-17_zam999117915s1.pdf]

Supplementary materials

Table S1. Nucleotide sequences of primers used for site-directed mutagenesis

Directed mutation	Oligonucleotide sequences[1]
E166P	5'-TAACTGGGAAGAT**CCA**AACGAAGTCG-3'
S185P	5'-TAACCAAGATAAT**CCA**TTTGTAAGAGA-3'
T350P	5'-AGAATGTAGAGAG**CCT**ATGTGGGGACA-3'
V212T	5'-TAGAATCGACACT**ACA**CCAGAAGTCAAA-3'
V232T	5'-AGATTGTTATGCT**ACT**GGAGAAATCTTCA-3'

[1] Nucleotide sequences corresponding to the mutated amino acids are underlined and in bold.

Table S2. GH13 subfamily enzymes used in the phylogenetic analysis.

**Source**	**GenPept accession number**	**GH13 subfamily**
**Ciliates**		
Euplotes crassus	AGU13047.1	
Euplotes focardii	AGU13046.1	
Euplotes harpa	MMETSP0213-20121227 (https://portal.camera.calit2.net/gridsphere/gridsphere).	
Tetrahymena thermophila	EAS00610.1	
Paramecium tetraurelia	CAK74111.1	
Oxytricha trifallax	EJY73324.1	
**Bacteria**		
Bacillus subtilis	CAA23437.1	28
Bacillus stearothermophilus	AAA22235.2	5
Escherichia coli	AAN82828.1	19
Pseudoalteromonas haloplanktis 1	CAA41481.1	15
Pseudoalteromonas haloplanktis 2	WP_041454408.1	32
Streptococcus mutans	AAC35010.1	5
Streptomyces limosus	AAA88554.1	32
Thermoactinomyces vulgaris	CAA49465.1	21
Vibrio cholerae	AAF96758.1	19
Xanthomonas campestris	AAA27591.1	27
Saccharophagus degradans (bacterial-	ABD79837.1	19
like)	ABD79827.1	6
Saccharophagus degradans (plant-like)		
**Archaea**	AAB67705.1	7
Pyrococcus furiosus	AAC97877.1	7
Thermococcus hydrothermalis		
**Fungi and yeast (GH13_1)**	P56271 (Swiss Prot)	1
Aspergillus niger	AAA32708.1	1
Aspergillus oryzae	CAA29233.1	1
Saccharomycopsis fibuligera	BAA12010.1	1
Cryptococcus sp. S-2	CAK40249.1	1
Aspergillus niger AgtA	CAK41088.1	1

Aspergillus niger AgtB		
**Fungi (GH13_5)**	AAW44866.1	5
Cryptococcus neoformans	ABK62854.1	5
Histoplasma capsulatum	CAK37367.1	5
Aspergillus niger-AmyD	CAK40250.1	5
Aspergillus niger-AmyE	EAA63277.1	5
Aspergillus nidulans	EAL90846.1	5
Aspergillus fumigatus	BAE56147.1	5
Aspergillus oryzae 1	BAE58539.1	5
Aspergillus oryzae 2	XP_959864.3	5
Neurospora crassa		
**Plants**	AAA98790.1	6
Hordeum vulgare	AAF63239.1	6
Malus domestica	BAA33879.1	6
Phaseolus vulgaris		
**Animals**	CAA28238.1	15
Drosophila melanogaster	AAA52279.1	24
Homo sapiens	CAA54524.1	24
Litopenaeus vannamei		

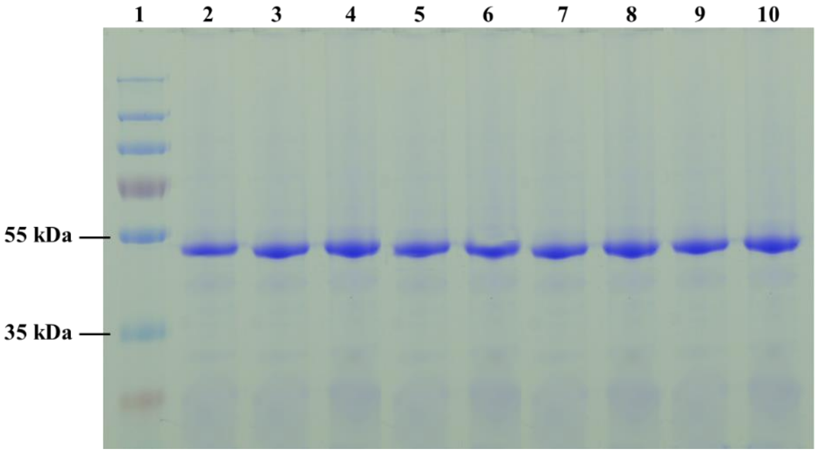

Fig. S1. SDS-PAGE of recombinant α-amylases. Wild-type *Ef*Amy (lane 2), E166P; (lane 3), S185P (lane 4), T350P (lane 5), V212T (lane 6), V232T (lane 7), V212T/V232T (lane 8), E166P/S185P/T350P (lane 9), E166P/S185P/T350P/V212T/V232T (lane 10). The protein ladder with respective molecular weights is shown in lane 1.

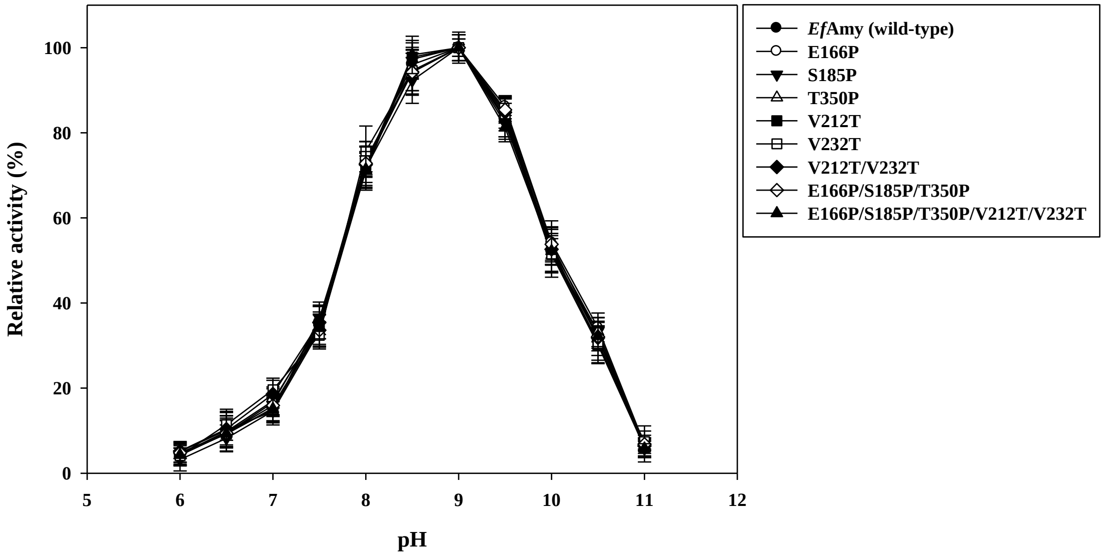

Fig. S2. Effect of pH on the amylolytic activity of *Ef*Amy and mutants.
